# Supplementary figures and images for: CD81 suppresses NF-κB signaling and is downregulated in hepatitis C virus expressing cells
Source: Front Cell Infect Microbiol. 2024 Jan 31;14:1338606. doi: 10.3389/fcimb.2024.1338606 (PMC10864554; doi:10.3389/fcimb.2024.1338606)

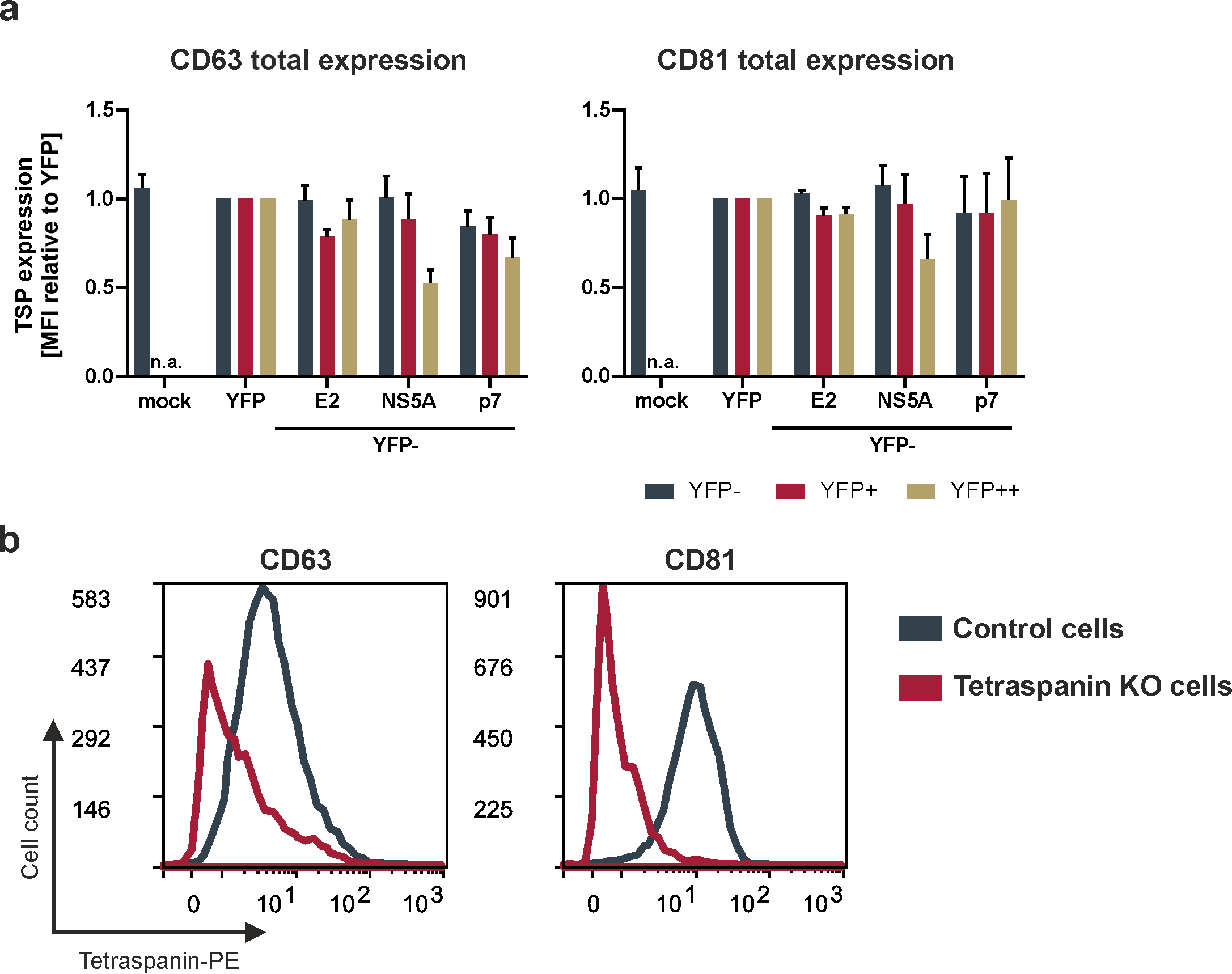

Supplement: Supplementary Figure 1 — Downregulation of tetraspanins by HCV proteins and characterization of tetraspanin knock-out cells. (A) Total cellular tetraspanin levels in cells transfected to express different viral proteins. HEK293T cells were transfected to express YFP-tagged HCV proteins. 24h after transfection cells were harvested for flow cytometric analysis, permeabilized and stained for total expression of tetraspanins CD63 and CD81. Shown are total cellular tetraspanin levels of cells expressing no (YFP-) to medium (YFP+) and high (YFP++) levels of viral proteins. Data from 3 independent biological replicates. (B) Analysis of Crispr control and tetraspanin knock-out Huh7.5 cells for CD63 and CD81 cell surface expression via flow cytometry. [file Image_1.tif]

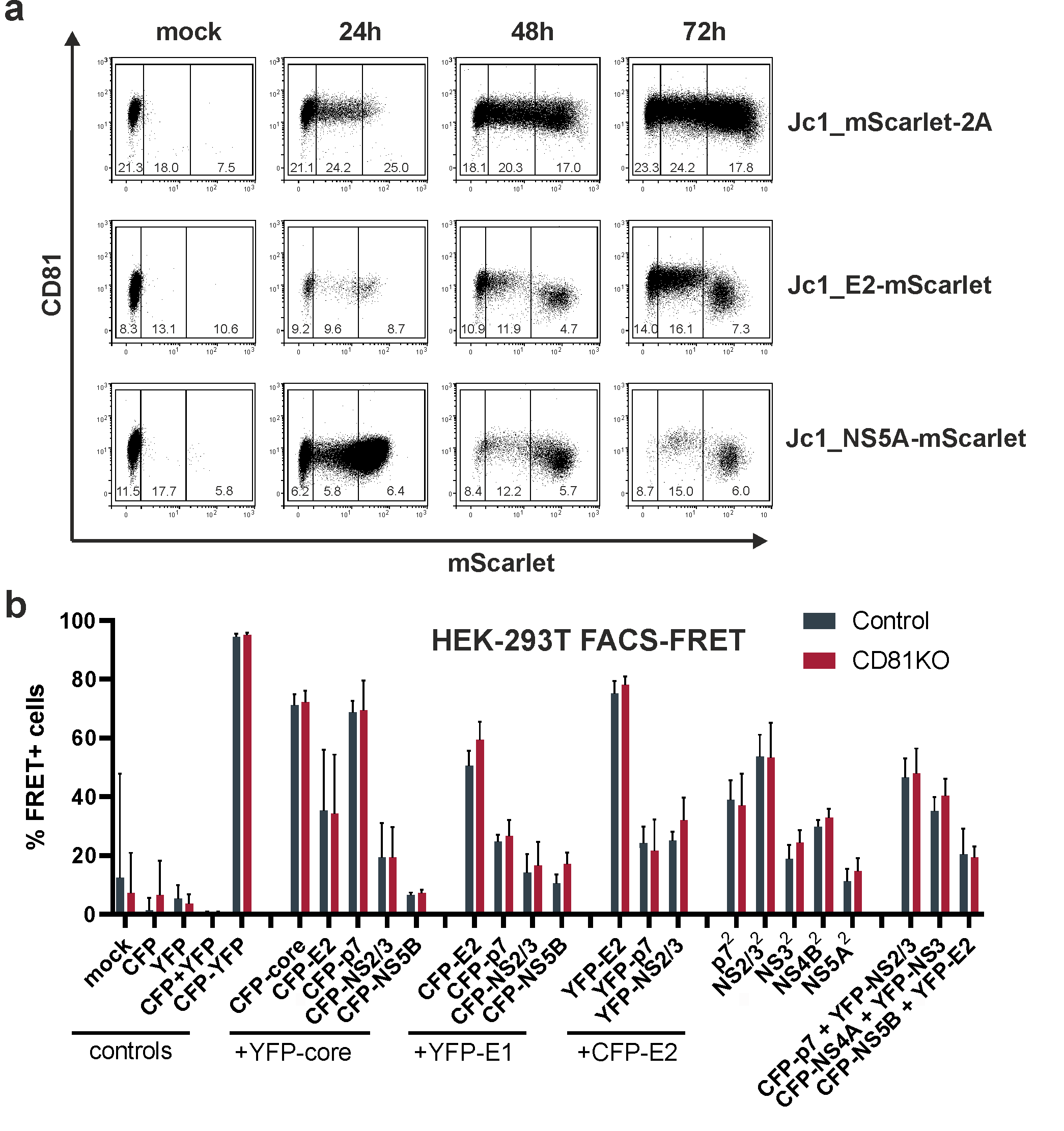

Supplement: Supplementary Figure 2 — Reduction of total CD81 in HCV-expressing cells and intraviral HCV protein interaction in CD81KO cells. (A) Representative experiment showing reduction in total CD81 levels by different viral genomes. Huh7.5 cells were electroporated with indicated viral genome RNAs and harvested for flow cytometric analysis at the indicated time points. Cells were permeabilized and stained for CD81. Gates show bystander (mSc-; left gate), medium (mSc+; middle gate) and high (mSc++; right gate) HCV expressing cells. Numbers within gates give the mean fluorescent intensity (MFI) of PE-CD81. (B) Interaction network of HCV proteins in presence or absence of CD81. HEK293T Crispr control and CD81KO cells were transfected to express a pair of eCFP- and eYFP-tagged viral proteins. 24h after transfection, cells were harvested and FRET signals were measured via flow cytometry as described (Hagen et al., 2014). Data from 4-8 independent biological replicates. Shown are mean values ± SD. [file Image_2.tif]

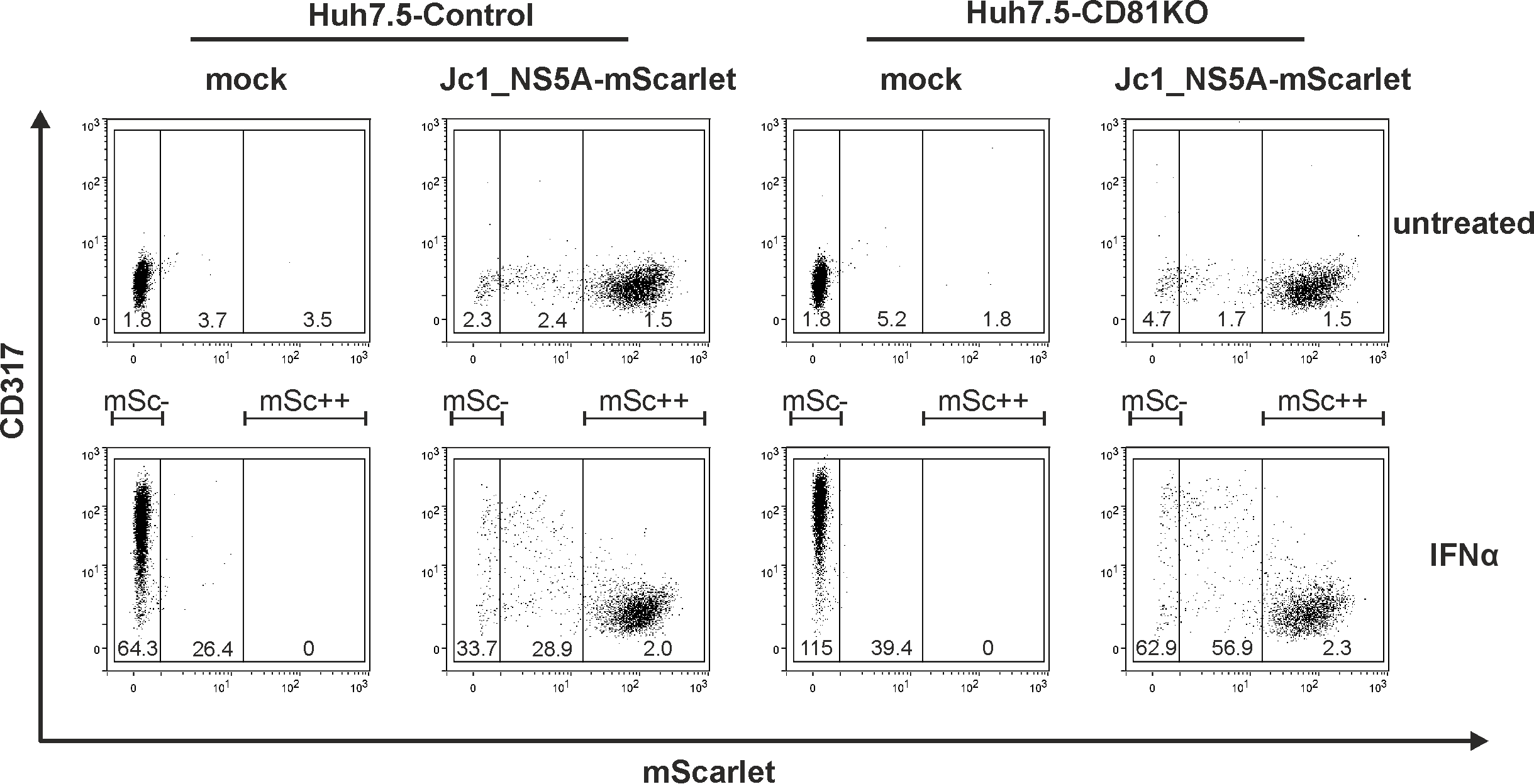

Supplement: Supplementary Figure 3 — CD81 does not impair the ability of HCV to counteract the interferon response. Representative experiment showing FACS plots of HCV expressing cells treated with IFNα, see also . Huh7.5 Crispr control and CD81KO cells were electroporated with Jc1_NS5A-mScarlet. 48h after EP cells were treated with IFNα (10ng/ml) for additional 24h, then harvested for flow cytometry and stained for surface expression of the ISG tetherin (CD317) as proxy for the interferon response. Gates show bystander cells (mSc-; left gate), and cells that express high (mSc++; right gate) levels of NS5A-mScarlet. Numbers within gates represent mean fluorescence intensity (MFI) of tetherin (PE-CD317) expression. [file Image_3.tif]
